# NGF promotes cell cycle progression by regulating D-type cyclins via PI3K/Akt and MAPK/Erk activation in human corneal epithelial cells

**Published:** 2012-03-30

**Authors:** Jiaxu Hong, Tingting Qian, Qihua Le, Xinghuai Sun, Jihong Wu, Junyi Chen, Xiaobo Yu, Jianjiang Xu

**Affiliations:** 1 Department of Ophthalmology, Eye, Ear, Nose, and Throat Hospital, School of Shanghai Medicine, Fudan University, Shanghai, China; 2 State Key Laboratory of Medical Neurobiology, Institutes of Brain Science, Shanghai, China

## Abstract

**Purpose:**

Nerve growth factor (NGF) plays an important role in promoting the healing of corneal wounds. However, the molecular mechanism by which NGF functions is unknown. We investigated the possible effects of NGF on phosphatidylinositol 3-kinase (PI3K)/protein kinase B (Akt) and mitogen activated protein kinase (MAPK)/extracellular signal-regulated kinase (Erk) pathways and cell growth in human corneal epithelial cells (HCECs).

**Methods:**

We examined the effect of NGF on cell cycle and proliferation in HCECs by flow cytometry and cell proliferation assay, respectively. The levels of D-type cyclins in NGF-treated HCECs were determined by western blot. The tyrosine kinase A (TrkA), PI3K/Akt and MAPK/Erk pathways were then checked in cells stimulated with NGF for different time periods or cells undergoing a dose-dependent treatment. Furthermore, HCECs were treated with pathway inhibitors, LY294002 or PD98059, to confirm NGF-induced activations.

**Results:**

We found that NGF had a positive effect on the growth of HCECs, and D-type cyclins, and it was correlated with the percentage of the G_1_ to S progression. We also observed a time-dependent and dose-dependent effect of NGF on the PI3K/Akt and MAPK/Erk pathways. Furthermore, NGF affected cell cycle progression of HCECs by regulating cyclin D through Akt and Erk activation upon treatment with the pathway inhibitors, LY294002 for Akt or PD98059 for Erk pathways.

**Conclusions:**

NGF stimulation could promote cell proliferation and cell cycle progression of HCECs by activation of cyclin D via the PI3K/Akt and MAPK/Erk signaling pathways.

## Introduction

Nerve growth factor (NGF), which is a well characterized factor of the neurotrophin family, plays an important role in growth, differentiation, and survival of neurons [1-4]. A similar role of NGF is also implicated in the regulation of corneal biologic functions. Previous studies have shown that NGF plays a pivotal role in modulating wound-healing processes in the cornea [5-10], and in vitro, NGF was found to induce the proliferation and differentiation of corneal epithelium [6]. Furthermore, Qi et al. [11] demonstrated that NGF might support cell self-renewal of human corneal epithelial progenitor cells. However, the molecular mechanisms by which NGF functions in corneal epithelium are still unclear.

NGF exerts its functions through two membrane receptors: the tyrosine kinase receptor (TrkA), a high-affinity receptor of NGF; and the neurotrophin receptor (p75NTR), a low-affinity and common receptor for all neurotrophins. NGF binds to TrkA to induce auto-phosphorylation of the TrkA receptor, leading to the activation of various signaling pathways, including phosphatidylinositol 3-kinase (PI3K)/protein kinase B (Akt), mitogen activated protein kinase (MAPK)/extracellular signal-regulated kinase (Erk) and phospholiase C-γ (PLC-γ) [12,13]. The PI3K/Akt and MAPK/Erk signaling pathways were found to be activated by insulin in HCECs [14], and the insulin-induced Erk pathway is involved in cell migration in human corneal epithelial cells (HCECs), leading to corneal wound healing. The PI3K/Akt signaling pathway is also activated by adenovirus type 19 infection to promote corneal cell survival [15]. Furthermore, PI3K/Akt as well as MAPK/Erk signaling has been identified to participate in the epidermal growth factor receptor related regulation of HCEC proliferation, migration [16]. These studies suggest that the PI3K/Akt and MAPK/Erk signaling pathways play an important part in regulating corneal biology. Although NGF has been widely reported to induce Akt and Erk activity in several types of cells [17-19], there are no relevant studies in HCECs.

Therefore, we investigated the effects of NGF on several major signaling pathways and cell growth in HCECs. To determine the growth regulatory mechanism of NGF, we examined the effect of NGF on the cell cycle in HCECs. We found that NGF promotes cell growth and G_1_-S transition by upregulation of D-type cyclin expression via activation of the Akt and Erk signaling pathways. Our data suggest that NGF regulates the cell cycle as an activator of Akt and Erk in HCECs, and might be a potential therapeutic factor in human corneal wound healing.

## Methods

### Cell culture

The Medical Ethics Committee of Shanghai Eye, Ear, Nose and Throat (EENT) Hospital approved the study protocol. The study was conducted in compliance with the Declaration of Helsinki. As described previously [6], corneoscleral rims from the donor corneas were obtained from the eye bank of Shanghai EENT Hospital (Shanghai, China) as soon as the central corneal button had been used for the penetrating keratoplasty. The tissue was rinsed with sodium chloride for three times and then treated with 1.3 unit/ml Dispase II in defined keratinocyte serum free medium (K-SFM; GIBCO, Grand Island, NY) at 4 °C for 16 h. The corneal epithelial sheets were peeled off and digested with 0.05% trypsin/0.02% EDTA at 37 °C for 10 min to be rendered into single cells which were then seeded on the 3T3 fibroblasts feeder layers. The 3T3 fibroblasts were treated with 10 μg/ml mitomycin C for 2 h and seeded in dishes. The HCECs used in the whole study was obtained from corneoscleral rims and cultured to passage one for further studies. HCECs were maintained in the medium composed by 75% K-SFM and 25% Dulbecco’s modified essential medium (DMEM) F-12 (Hyclone; Thermo, Fisher Scientific Inc., Logan, UT), The DMEM F-12 was supplemented with 10% fetal bovine serum (GIBCO) and 1% penicillin/streptomysin (Ji Nuo Biologic Science Co. Ltd, Shanghai, China). The cells used for the experiments except cell proliferation assay or cell cycle analysis were incubated in define K-SFM without growth factors for 24 h for synchronization. Before harvest for the analysis, the cells were treated with 0.25% trypsin/0.02% EDTA at room temperature to remove the 3T3 fibroblasts feeder layers.

### Drug treatment

For drug treatment, cells were incubated in K-SFM without growth factors for 24 h for synchronization, and then treated for the indicated concentration and period. Drugs were used as follows: human recombinant NGF-β from Sigma-Aldrich (St. Louis, MO), diluted in PBS containing 0.1% BSA; the PI3K/Akt inhibitor LY294002; and the MAPK/ Erk inhibitor PD98059 from Sigma-Aldrich, diluted in dimethyl sulfoxide.

### Immunostaining

HCECs cultured in 6-well plates were fixed in 95% ethanol for 20 min and then dried at room temperature. After three rinses with PBS for 5 min each and pre-incubation with 1% normal rabbit serum to block nonspecific staining, the cells were then incubated with anti-cytokeratin 12 antibody (1:100) from Santa Cruz Biotechnology (Santa Cruz, CA) for 1 h. After three washes with PBS for 5 min each, cells were incubated with a FITC-conjugated secondary antibody (1:100; Sigma-Aldrich, St. Louis, MO) for 45 min. Cells were then washed for three additional times, and counterstained with Hoechst 33342 (10 g/ml). Mounted with a Mowiol 4–88 media (Sigma-Aldrich), HCECs were analyzed with a fluorescence microscope.

### Cell proliferation assay

The CellTiter 96^®^ AQueous One Solution cell proliferation assay kit (Promega, Madison, WI) was used for measurement of cell proliferation. As described by the manufacturer, cells were seeded onto 96-well plates at the density of 6×10^3^ per well in defined K-SFM with or without the recombinant NGF-β and then incubated with 20 µl/well CellTiter 96^®^ AQueous One Solution Reagent at 37 °C for 4 h. The absorbance was recorded at 490 nm using a 96-well plate reader (Bio-Rad, CA).

### Cell cycle analysis

Cells were trypsinized, washed once with ice-cold PBS, and fixed with 75% ethanol at −20 °C overnight. After washing twice with PBS, cells were stained with 10 µg/ml propidium iodide (Sigma-Aldrich) containing 1 mg/ml RNase A (Sigma-Aldrich) at 37 °C for 20 min in the dark and analyzed with FACSCalibur flow cytometer and CellQuest software (BD Biosciences, San Diego, CA).

### Immunoblotting

Cells were lysed in radioimmunoprecipitation assay (RIPA) buffer supplemented with protease inhibitors (Complete, Mini, EDTA-free; Roche Applied Science, Indianapolis, IN) and phosphatase inhibitor cocktails 1 and 2 (Sigma) and centrifuged to get the cleared lysates. Antibodies against Tyr674/675 phospho-TrkA, total TrkA, Ser473 phospho-Akt, total Akt, Thr202/Tyr204 phospho-p44/42 MAPK (Erk1/2), total p44/42 MAPK (Erk1/2), cyclin D2, and anti-Rabbit IgG HRP-conjugated Secondary Antibody were from Cell Signaling Technology (Beverly, MA). Antibody against cyclin D1 was from Santa Cruz Biotechnology (Santa Cruz, CA). Antibody against glyceraldehyde phosphate dehydrogenase (GAPDH) was the gift from Miao Tong Biologic Science & Technology Co. Ltd (Shanghai, China).

### Reverse transcription-PCR

Total RNA was isolated with Trizol (Invitrogen, Carlsbad, CA) and reverse transcription-PCR (RT–PCR) was done with RT–PCR system (TAKARA Biotechnology, Dalian, China) according to the instructions of the manufacturers. Amplified cDNAs were then performed for PCR. The primers used are listed in Table 1.

**Table 1:** RT–PCR primers.

**Gene**	**Direction**	**Primer sequence**
cyclin D1	Forward	AGCCATGGAACACCAGCTC
	Reverse	GCACCTCCAGCATCCAGGT
cyclin D2	Forward	TACTTCAAGTGCGTGCAGAAGGAC
	Reverse	TCCCACACTTCCAGTTGCGATCAT
*GAPDH*	Forward	GCCAAGGTCATCCATGACAAC
	Reverse	GTCCACCACCCTGTTGCTGTA

## Results

### NGF promotes cell growth of human corneal epithelial cells

**Figure 1:**
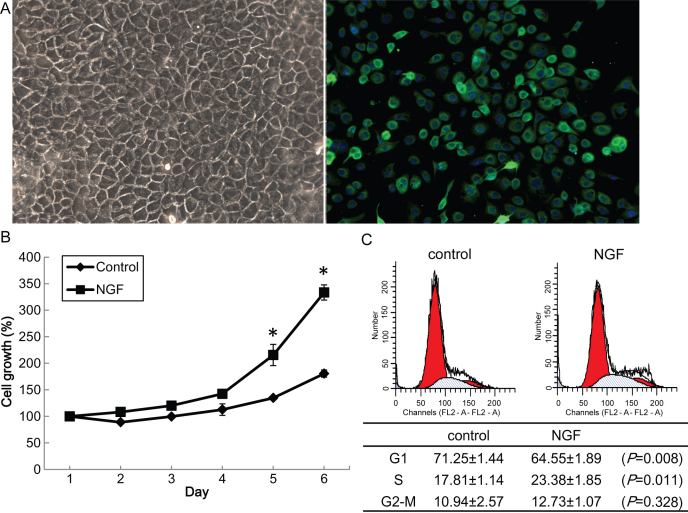
Effect of nerve growth factor on cell proliferation and the cell cycle in human corneal epithelial cells. **A**: Human corneal epithelial cells (HCECs) cultured in vitro displayed a polygonal pattern (left panel). Immunostaining for cytokeratin 12 (green) followed by Hoechst staining (blue; right panel) is shown. **B**: HCECs were seeded onto 96 well plates at a density of 6×10^3^ per well in defined keratinocyte serum-free medium (K-SFM), and then treated with recombinant nerve growth factor β-NGF at 5 ng/ml and subjected to a cell proliferation assay for up to 6 days. **C**: HCECs at passage 1 were plated onto 60-mm dishes. The cell cycle was analyzed by flow cytometry with cells treated identically at day 4 of the cell proliferation assay (upper panel), and the percentage of each phase (G_1_-M) is indicated on the right (lower panel). Experiments were performed in triplicate and statistically analyzed with the Student *t* test. The results are shown as mean±standard deviation (SD). All p-values (*) were considered statistically significant when p<0.05.

HCECs cultured in vitro displayed a polygonal pattern, and expressed cytokeratin 12, which is a biomarker for corneal epithelium (Figure 1A). To explore the role of NGF in HCECs, we first performed a cell proliferation assay to analyze cell growth. As shown in Figure 1B, HCECs were treated with human recombinant β-NGF in defined K-SMF with growth factors, and cell growth was promoted by NGF treatment at 5 ng/ml (p<0.05, Student’s *t* test). HCECs were then treated with NGF and the cell cycle was analyzed by flow cytometry with cells treated identically as day 4 of the cell proliferation assay. The percentage of G_1_ phase cells was slightly reduced in NGF treated cells, while the S phase cells were increased (p<0.05, Student’s *t* test, Figure 1C). Taken together, these results indicate that NGF accelerated growth of human corneal epithelial cells through G_1_ promotion.

### NGF induces activity of the PI3K/Akt and MAPK/Erk signaling pathways in HCECs

**Figure 2:**
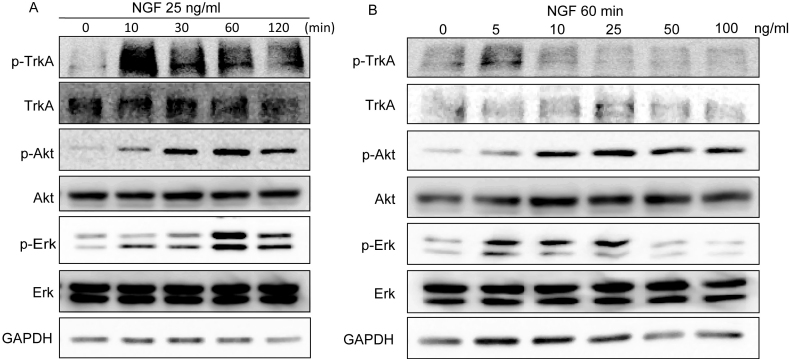
Effect of nerve growth factor on the phosphatidylinositol 3-kinase (PI3K)/protein kinase B (Akt) and mitogen activated protein kinase (MAPK)/extracellular signal-regulated kinase (Erk) signaling pathways in human corneal epithelial cells. **A**: Human corneal epithelial cells (HCECs) were incubated in defined keratinocyte serum-free medium (K-SFM) without growth factors for 24 h before nerve growth factor (NGF) stimulation and then treated with NGF at 25 ng/ml for different periods. **B**: HCECs were treated with different concentrations of NGF for 60 min. A total of 50 μg cell lysates were analyzed for expression of the indicated genes by immunoblotting analysis. Tyr674/675 phospho-TrkA indicates the NGF signaling activity. Glyceraldehyde 3-phosphate dehydrogenase (GAPDH) was used as a loading control. For both **A** and **B**, results are representative of three independent experiments. Experiments were performed in triplicate.

Since we found that cell growth of HCECs was positively regulated by NGF, we then investigated the mechanism by which NGF enhanced HCEC proliferation. To determine the optimal conditions of NGF treatment for the mechanism study, we treated HCECs with various concentration of NGF and examined at different time points, and measured the activity of NGF signaling. TrkA, the high-affinity receptor of NGF, was activated immediately when cells were stimulated by NGF (Figure 2A). Although NGF has been shown to regulate several signaling pathways in other cell lines, it is still unclear whether NGF affects signaling pathways in HCECs. Therefore, we examined two important downstream signaling pathways, PI3K/Akt and MAPK/Erk, which play important roles in cell proliferation. In the time-dependent treatment, phosphorylation levels of Akt (p-Akt) at Ser473 and Erk1/2 (p-Erk) at Thr202/Tyr204 were increased within 10 min, reached a maximum after 60 min and remained high even after 2 h (Figure 2A). Upon dose-dependent treatment, NGF caused increased phosphorylation of the Akt and Erk pathways at a concentration of 5 ng/ml and a maximal effect was observed at 25 ng/ml (Figure 2B). However, the Akt and Erk pathways underwent delayed activation that lagged behind the phosphorylation of TrkA. These results indicated that NGF induced the activity of the Akt and Erk signaling pathways in HCECs, which may require time for signaling transmission.

### NGF promotes cell cycle progression via Akt and Erk activation in HCECs

**Figure 3:**
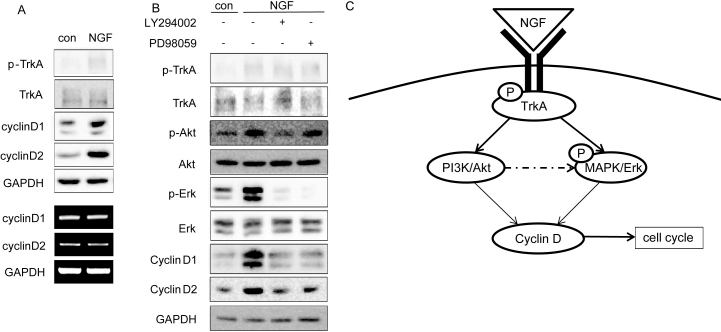
Nerve growth factor promotes cell cycle progression via Akt and Erk activation in human corneal epithelial cells. **A**: Human corneal epithelial cells (HCECs) were incubated in keratinocyte serum-free medium (K-SFM) without growth factors for 24 h before treatment with nerve growth factor (NGF) at 25 ng/ml for 1 h. A total of 50 µg cell lysates and total RNA were analyzed for expression of the indicated genes by western blot analysis and reverse transcriptase–polymerase chain reaction (RT–PCR), respectively. Glyceraldehyde 3-phosphate dehydrogenase (GAPDH) was used as a loading control. **B**: HCECs were incubated in K-SFM without growth factors for 24 h and then pretreated with LY294002 or PD98059 at 10 µM for 1 h before treatment with NGF at 25 ng/ml for another hour. A total of 50 μg cell lysates were analyzed for expression of the indicated genes by immunoblotting analysis. GAPDH was used as a loading control. Experiments were performed in triplicate. **C**: The schematic representation depicts how the Akt and Erk pathways collaborate to control NGF induction of cyclin D expression.

According to our results shown in Figure 2, we treated HCECs at 25 ng/ml for 1 h and examined the expression of D-type cyclin, which play crucial roles in G_1_ to S transition. We found that both cyclin D1 and cyclin D2 expression were upregulated by NGF at the protein level but not the mRNA level in HCECs (Figure 3A). Since we found that the Akt and Erk pathways were positively regulated by NGF at the phosphorylation level, we next investigated whether there was any correlation between the signaling pathways and NGF-induced cell growth. We found that the phosphorylation of Erk was markedly repressed with treatment with the PI3K/Akt inhibitor LY294002 and MAPK/Erk inhibitor PD98059 (Figure 3B). LY294002-treated HCECs displayed a significant decrease in Akt and Erk phosphorylation, while PD98059-treated cells showed a decrease in the Erk phosphorylation, which suggested that Akt pathway might be the upstream of the Erk pathway after NGF treatment. Furthermore, both D-type cyclins expressions were repressed by LY294002 or PD98059 treatment, which suggested that cyclin D was the downstream target of the Akt and Erk pathways. Therefore, these data showed a positive effect of NGF on the Akt and Erk pathways, and suggested that NGF promoted cell cycle progression of HCECs through the Akt and Erk pathways.

## Discussion

In this study, we showed that NGF stimulation might favor cell cycle progression of human corneal epithelial cells by activation of cyclin D via the Akt and Erk signaling pathways. Our data also indicated that activation of the Akt or Erk signaling pathway by NGF was time- and dose-dependent. This positive effect of NGF on cell growth of HCECs was confirmed by treating cells with inhibitors of these signaling pathways, namely LY294002 for Akt and PD98059 for Erk.

Previous studies have shown that corneal nerve injury results in the dysfunction of corneal epithelium and delays its wound healing [7,20], and NGF is indispensable for corneal wound healing. Autocrine NGF levels of the corneal epithelium are significantly increased in the wounded eye after 24 h [6]. The wound healing rate is decreased when NGF is down-regulated by bevacizumab eye drops [21]. After laser-assisted in situ keratomileusis (LASIK), commonly referred to as laser eye surgery, which cuts off the corneal nerve, autocrine NGF levels in tears are decreased [22] and the loss of innervation contributes to the loss of keratocytes after LASIK [23]. NGF eye-drops can promote the healing of corneal ulcers [7,24], and can be a therapeutic factor in preventig allograft rejection in the cornea. NGF treatment may restore a deficit in the synthesis or release of endogenous NGF [25,26]. Therefore, NGF might be involved in sensory innervation and proliferation and differentiation of epithelial cells [27]. However, the exact molecular mechanism of the effect of NGF on human corneal epithelium is not well defined. Our study revealed that NGF promotes cell cycle progression by regulating D-type cyclins via Akt activation in human corneal epithelial cells. It should be pointed out that HCECs were treated by NGF at 25 ng/ml for 1 h in the current study for cell growth or cell cycle analysis. This concentration is different from results of previous report. Bonini et al. found that murine NGF (100–200 mg/ml) improved corneal sensitivity and promoted corneal epithelial healing in both moderate and severe neurotrophic keratitis [7]. Kruse et al. studied two different concentrations of NGF (50 and 250 ng/ml) and their effects on growth and differentiation of rabbit limbal epithelium [28]. They found that the colony size of cells was significantly increased by NGF at the concentrations of 250 ng/ml compared with 50 ng/ml. The various concentrations and times of NGF treatment might be attributed to the difference between in vivo and in vitro testing, and different cultured cells (rabbit versus human). Therefore, the Akt and Erk pathways, which were previously reported to regulate cell growth, proliferation, differentiation and angiogenesis [29], are also activated by NGF in HCECs.

Cyclin D is a member of the cyclin-dependent kinase (Cdk) family of serine/threonine kinases, which play a crucial role in modulating progression through the cell cycle. Cyclin D1 has been identified to be induced concordant with NGF-induced neurite outgrowth in PC12 cells [30-32]. Cyclin D2 has also been reported to be positively correlated with NGF expression [33-35]. Moreover, Marampon et al. [31] suggested that cyclin D1 induction by NGF involves the PI3K/Akt, Erk and p38 pathways in PC12 cells. However, it is unknown whether NGF-induced corneal wound healing is correlated with expression of D-type cyclins. In our study, NGF promoted cell growth and enhanced cyclin D expression at the protein level, but not the mRNA level (Figure 3A), which is different from results in other studies that showed NGF stimulates cyclin D1 promoter activity and mRNA synthesis [30,31]. The reason for this difference between studies might be that the peak time point for protein and mRNA level might differ. Cyclin D1 is reported to be excluded from the nucleus during the S phase and the protein is rapidly degraded through phosphorylation by β-glycogen synthase kinase 3 [36]. Therefore, NGF might play a pivotal role in inhibiting the degradation of cyclin D by other factors. It is possible that NGF promotes cell cycle progression of HCECs through rescuing the expression of cyclin D.

The PI3K/Akt signaling pathway has been shown to promote corneal cell survival [15]. MAPK/Erk as well as PI3K/Akt also play a positive role in HCEC proliferation, migration and corneal wound healing [14,16]. In addition, our results of microarray analysis found that D-type cyclins were involved in the proliferation of HCECs upon NGF treatment. We therefore hypothesize that each of these signaling pathways contribute to NGF induction of cyclin D expression, and thereby to HCEC proliferation. In the current study, we shownd that NGF-induced cyclin D protein expression was strongly reduced by chemical inhibitors of the PI3K/Akt (LY294002) and MAPK/Erk (PD098059) pathways, suggesting that NGF might regulate cyclin D1 and cyclin D2 expression via Akt and Erk (Figure 3). Upon NGF treatment, Akt pathway inhibition also reduced the Erk activation status. However, inhibition of the Erk pathway seemed to have very little effect on Akt phosphorylation levels, suggesting that the Erk signaling pathway might be downstream of Akt in the process of NGF-induced cell proliferation of HCECs (Figure 3B).

In conclusion, our findings demonstrate that activation of cyclin D via the PI3K/Akt and MAPK/Erk signaling pathways are involved in the proliferation of corneal epithelial cells treated with NGF. Further investigation is required to determine whether NGF is associated with self-renewal of corneal epithelial stem cells at the limbus, and if it is a potential regulator of the limbal stem cell niche.
